# *N*-Methyl-2-oxo-1-phenyl­propan-1-aminium chloride

**DOI:** 10.1107/S1600536811024032

**Published:** 2011-06-30

**Authors:** Shi Juan Wang

**Affiliations:** a Department of Applied Chemistry, Nanjing College of Chemical Technology, Nanjing 210048, People’s Republic of China

## Abstract

In the structure of the title compound, C_10_H_14_NO^+^·Cl^−^, both H atoms bound to nitro­gen are involved in N—H⋯Cl hydrogen-bonding inter­actions. These inter­actions join the cations and anions into dimeric units (two cations and two anions) with *R*_4_^2^(8) motifs lying about inversion centers.

## Related literature

For the screening of mol­ecular salts with physicochemical properties, see: Tong & Whitesell *et al.* (1998 ▶); Shanker (1994 ▶). Over 40% of commercially available salts are hydro­chlorides (Gould *et al.*, 1986 ▶), and this trend is reflected in the available set of salt structures included in the Cambridge Structural Database (Allen *et al.*, 2002 ▶). For a closely related structure, see: Au & Tafeenko (1986 ▶). 
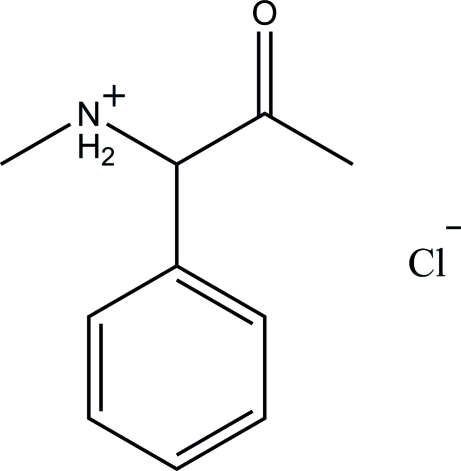


## Experimental

### 

#### Crystal data

C_10_H_14_NO^+^·Cl^−^
*M* *_r_* = 199.67
Monoclinic, 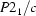

*a* = 12.631 (3) Å
*b* = 8.2564 (17) Å
*c* = 11.423 (2) Å
β = 114.63 (3)°
*V* = 1082.9 (4) Å^3^
*Z* = 4
Mo *K*α radiation
μ = 0.32 mm^−1^
*T* = 293 K
0.20 × 0.20 × 0.20 mm

#### Data collection

Rigaku Mercury 2 diffractometer
Absorption correction: multi-scan (*CrystalClear*; Rigaku, 2002 ▶) *T*_min_ = 0.825, *T*_max_ = 1.000
10899 measured reflections
2486 independent reflections
1858 reflections with *I* > 2σ(*I*)
*R*_int_ = 0.053

#### Refinement

*R*[*F* ^2^ > 2σ(*F* ^2^)] = 0.053
*wR*(*F* ^2^) = 0.172
*S* = 1.12
2486 reflections
118 parameters
H-atom parameters constrained
Δρ_max_ = 0.27 e Å^−3^
Δρ_min_ = −0.20 e Å^−3^

### 

Data collection: *CrystalClear* (Rigaku, 2002 ▶); cell refinement: *CrystalClear*; data reduction: *CrystalClear*; program(s) used to solve structure: *SHELXS97* (Sheldrick, 2008 ▶); program(s) used to refine structure: *SHELXL97* (Sheldrick, 2008 ▶); molecular graphics: *DIAMOND* (Brandenburg, 1998 ▶); software used to prepare material for publication: *SHELXTL* (Sheldrick, 2008 ▶).

## Supplementary Material

Crystal structure: contains datablock(s) I, global. DOI: 10.1107/S1600536811024032/yk2004sup1.cif

Structure factors: contains datablock(s) I. DOI: 10.1107/S1600536811024032/yk2004Isup2.hkl

Supplementary material file. DOI: 10.1107/S1600536811024032/yk2004Isup3.cml

Additional supplementary materials:  crystallographic information; 3D view; checkCIF report

## Figures and Tables

**Table 1:** Hydrogen-bond geometry (Å, °)

*D*—H⋯*A*	*D*—H	H⋯*A*	*D*⋯*A*	*D*—H⋯*A*
N1—H1*A*⋯Cl1	0.90	2.26	3.1345 (19)	163
N1—H1*E*⋯Cl1^i^	0.90	2.19	3.0747 (19)	167

Symmetry code: (i) 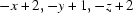.

**Fig. 1.**
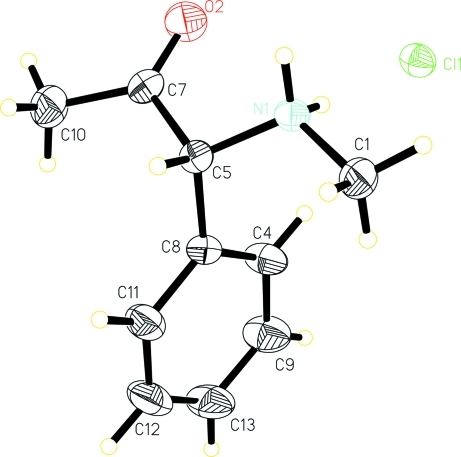
View of the asymmetric unit of the title compound, showing the atom-numbering scheme. Displacement ellipsoids are drawn at the 30% probability level.

**Fig. 2.**
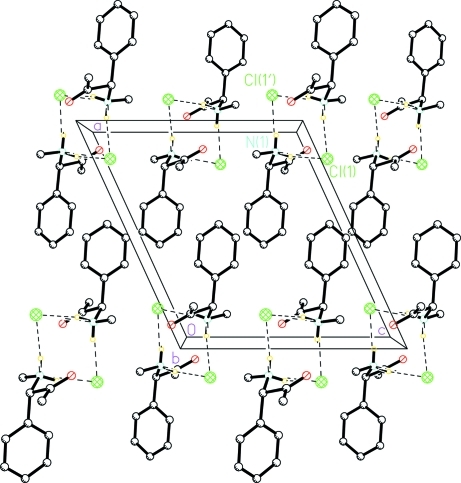
The crystal packing of the title compound viewed along the b axis showing the hydrogen bonds N—H···Cl (dotted lines).

## supplementary crystallographic information

### Comment

The importance of molecular salts as solid forms in pharmaceutical formulations is well known. For a given active ingredient, the isolation and selection of a salt with the appropriate physicochemical properties involves significant screening activity and has been discussed at some length in the literature (Tong & Whitesell *et al.*, 1998; Shanker *et al.*, 1994). It is apparent that over 40% of marketed salts are hydrochlorides (Gould *et al.*, 1986), and this trend is reflected in the available set of salt structures provided by the Cambridge Structural Database (Allen *et al.*, 2002). Here we report the synthesis and crystal structure of the title compound, *N*-methyl-2-oxo-1-phenylpropan-1-aminium chloride (Fig. 1).

The bond distances and angles in the structure of the title compound agree very well with the corresponding distances and angles reported for a closely related compound (Au & Tafeenko *et al.*, 1986). It is noteworthy that both H-atoms bonded to one nitrogen (N1) are involved in hydrogen bonding interactions of the type N—H···Cl hydrogen bonds, forming dimers lying about inversion centers according to *R*_2_^2^(4) motifs in graph set notation (Tab.1, Fig.2). Dipole-dipole and van der Waals interactions are effective in the molecular packing.

### Experimental

To a stirred solution of 1-(methylamino)-1-phenylpropan-2-one (2.445 g, 0.015 mol) in 30 mL of dry THF, hydrochloric acid (1.52 g, 0.015 mol) was added at the room temperature. The precipitate was filtered and washed with a small amount of ethanol 95%. Single crystals suitable for X-ray diffraction analysis were obtained from slow evaporation of a solution of the title compound in water at room temperature.

### Refinement

The H-atoms bonded to the C-atom were positioned geometrically and refined using a riding model, with C—H = 0.93–0.97 Å and *U*_iso_(H) = 1.2*U*_eq_(C). The H-atoms bonded to the N-atom were located from a difference map and refined using a riding model.

### Crystal data

**Table:**

C_10_H_14_NO^+^·Cl^−^	*F*(000) = 424
*M**_r_* = 199.67	*D*_x_ = 1.225 Mg m^−^^3^
Monoclinic, *P*2_1_/*c*	Mo *K*α radiation, λ = 0.71073 Å
Hall symbol: -P 2ybc	Cell parameters from 2486 reflections
*a* = 12.631 (3) Å	θ = 2.6–27.5°
*b* = 8.2564 (17) Å	µ = 0.32 mm^−^^1^
*c* = 11.423 (2) Å	*T* = 293 K
β = 114.63 (3)°	Prism, colorless
*V* = 1082.9 (4) Å^3^	0.20 × 0.20 × 0.20 mm
*Z* = 4	

### Data collection

**Table:**

Rigaku Mercury 2 diffractometer	2486 independent reflections
Radiation source: fine-focus sealed tube	1858 reflections with *I* > 2σ(*I*)
graphite	*R*_int_ = 0.053
Detector resolution: 13.6612 pixels mm^-1^	θ_max_ = 27.5°, θ_min_ = 3.0°
CCD_Profile_fitting scans	*h* = −16→16
Absorption correction: multi-scan (*CrystalClear*; Rigaku, 2002)	*k* = −10→10
*T*_min_ = 0.825, *T*_max_ = 1.000	*l* = −14→14
10899 measured reflections	

### Refinement

**Table:**

Refinement on *F*^2^	Primary atom site location: structure-invariant direct methods
Least-squares matrix: full	Secondary atom site location: difference Fourier map
*R*[*F*^2^ > 2σ(*F*^2^)] = 0.053	Hydrogen site location: inferred from neighbouring sites
*wR*(*F*^2^) = 0.172	H-atom parameters constrained
*S* = 1.12	*w* = 1/[σ^2^(*F*_o_^2^) + (0.1*P*)^2^ + 0.0*P*] where *P* = (*F*_o_^2^ + 2*F*_c_^2^)/3
2486 reflections	(Δ/σ)_max_ = 0.001
118 parameters	Δρ_max_ = 0.27 e Å^−^^3^
0 restraints	Δρ_min_ = −0.20 e Å^−^^3^

### Special details

**Table:**

Geometry. All e.s.d.'s (except the e.s.d. in the dihedral angle between two l.s. planes) are estimated using the full covariance matrix. The cell e.s.d.'s are taken into account individually in the estimation of e.s.d.'s in distances, angles and torsion angles; correlations between e.s.d.'s in cell parameters are only used when they are defined by crystal symmetry. An approximate (isotropic) treatment of cell e.s.d.'s is used for estimating e.s.d.'s involving l.s. planes.
Refinement. Refinement of *F*^2^ against ALL reflections. The weighted *R*-factor *wR* and goodness of fit *S* are based on *F*^2^, conventional *R*-factors *R* are based on *F*, with *F* set to zero for negative *F*^2^. The threshold expression of *F*^2^ > σ(*F*^2^) is used only for calculating *R*-factors(gt) *etc*. and is not relevant to the choice of reflections for refinement. *R*-factors based on *F*^2^ are statistically about twice as large as those based on *F*, and *R*- factors based on ALL data will be even larger.

### Fractional atomic coordinates and isotropic or equivalent isotropic displacement parameters (Å^2^)

**Table:**

	*x*	*y*	*z*	*U*_iso_*/*U*_eq_	
Cl1	0.84648 (5)	0.60715 (7)	1.05463 (5)	0.0477 (2)	
N1	0.88712 (14)	0.4261 (2)	0.83566 (16)	0.0372 (4)	
H1A	0.8633	0.4628	0.8949	0.045*	
H1E	0.9638	0.4049	0.8764	0.045*	
C4	0.6325 (2)	0.3894 (3)	0.7610 (2)	0.0557 (7)	
H4A	0.6721	0.4438	0.8384	0.067*	
C5	0.82470 (17)	0.2721 (2)	0.78105 (19)	0.0371 (5)	
H5A	0.8544	0.2286	0.7207	0.045*	
C7	0.85316 (19)	0.1511 (3)	0.8920 (2)	0.0429 (5)	
C8	0.69366 (18)	0.2949 (3)	0.7093 (2)	0.0396 (5)	
C9	0.5129 (2)	0.4023 (4)	0.6973 (3)	0.0725 (9)	
H9A	0.4724	0.4663	0.7317	0.087*	
C10	0.8142 (3)	−0.0187 (3)	0.8562 (3)	0.0646 (8)	
H10A	0.8363	−0.0829	0.9329	0.097*	
H10B	0.8501	−0.0618	0.8036	0.097*	
H10C	0.7311	−0.0212	0.8091	0.097*	
C11	0.6337 (2)	0.2185 (3)	0.5944 (2)	0.0606 (7)	
H11A	0.6737	0.1578	0.5574	0.073*	
C12	0.5143 (3)	0.2308 (4)	0.5332 (3)	0.0782 (10)	
H12A	0.4746	0.1759	0.4561	0.094*	
C13	0.4533 (2)	0.3214 (4)	0.5833 (3)	0.0747 (9)	
H13A	0.3727	0.3286	0.5412	0.090*	
C1	0.8697 (2)	0.5566 (3)	0.7395 (2)	0.0535 (6)	
H1B	0.9126	0.6511	0.7824	0.080*	
H1C	0.7884	0.5826	0.6970	0.080*	
H1D	0.8970	0.5202	0.6771	0.080*	
O2	0.90399 (18)	0.1954 (2)	1.00174 (16)	0.0685 (6)	

### Atomic displacement parameters (Å^2^)

**Table:**

	*U*^11^	*U*^22^	*U*^33^	*U*^12^	*U*^13^	*U*^23^
Cl1	0.0439 (4)	0.0545 (4)	0.0443 (4)	0.0016 (2)	0.0181 (3)	−0.0090 (2)
N1	0.0367 (10)	0.0430 (10)	0.0320 (9)	0.0007 (7)	0.0144 (8)	0.0012 (7)
C4	0.0402 (13)	0.081 (2)	0.0412 (14)	0.0042 (12)	0.0126 (11)	−0.0107 (12)
C5	0.0380 (11)	0.0403 (11)	0.0351 (11)	0.0030 (9)	0.0174 (9)	0.0010 (9)
C7	0.0411 (12)	0.0458 (12)	0.0444 (13)	0.0062 (10)	0.0206 (10)	0.0087 (10)
C8	0.0359 (11)	0.0461 (13)	0.0330 (11)	−0.0001 (9)	0.0106 (9)	0.0020 (9)
C9	0.0463 (15)	0.104 (3)	0.0649 (18)	0.0143 (15)	0.0204 (14)	−0.0088 (16)
C10	0.0814 (19)	0.0464 (15)	0.0628 (18)	−0.0013 (13)	0.0269 (15)	0.0096 (12)
C11	0.0575 (16)	0.0673 (17)	0.0466 (14)	0.0056 (13)	0.0114 (12)	−0.0156 (12)
C12	0.0593 (18)	0.091 (2)	0.0570 (17)	−0.0015 (16)	−0.0033 (14)	−0.0222 (16)
C13	0.0389 (15)	0.103 (2)	0.0652 (19)	0.0037 (15)	0.0045 (13)	−0.0044 (18)
C1	0.0651 (16)	0.0445 (13)	0.0481 (14)	−0.0040 (12)	0.0208 (12)	0.0077 (10)
O2	0.0939 (15)	0.0638 (13)	0.0361 (10)	0.0005 (10)	0.0155 (9)	0.0099 (8)

### Geometric parameters (Å, °)

**Table:**

N1—C1	1.488 (3)	C9—C13	1.375 (4)
N1—C5	1.489 (3)	C9—H9A	0.9300
N1—H1A	0.9000	C10—H10A	0.9600
N1—H1E	0.9000	C10—H10B	0.9600
C4—C9	1.380 (3)	C10—H10C	0.9600
C4—C8	1.391 (3)	C11—C12	1.376 (4)
C4—H4A	0.9300	C11—H11A	0.9300
C5—C8	1.522 (3)	C12—C13	1.359 (4)
C5—C7	1.534 (3)	C12—H12A	0.9300
C5—H5A	0.9800	C13—H13A	0.9300
C7—O2	1.203 (3)	C1—H1B	0.9600
C7—C10	1.486 (4)	C1—H1C	0.9600
C8—C11	1.366 (3)	C1—H1D	0.9600
			
C1—N1—C5	114.80 (17)	C4—C9—H9A	119.7
C1—N1—H1A	108.6	C7—C10—H10A	109.5
C5—N1—H1A	108.6	C7—C10—H10B	109.5
C1—N1—H1E	108.6	H10A—C10—H10B	109.5
C5—N1—H1E	108.6	C7—C10—H10C	109.5
H1A—N1—H1E	107.5	H10A—C10—H10C	109.5
C9—C4—C8	119.9 (2)	H10B—C10—H10C	109.5
C9—C4—H4A	120.0	C8—C11—C12	120.4 (3)
C8—C4—H4A	120.0	C8—C11—H11A	119.8
N1—C5—C8	112.75 (17)	C12—C11—H11A	119.8
N1—C5—C7	108.00 (17)	C13—C12—C11	121.3 (3)
C8—C5—C7	110.70 (17)	C13—C12—H12A	119.3
N1—C5—H5A	108.4	C11—C12—H12A	119.3
C8—C5—H5A	108.4	C12—C13—C9	118.9 (3)
C7—C5—H5A	108.4	C12—C13—H13A	120.6
O2—C7—C10	123.0 (2)	C9—C13—H13A	120.6
O2—C7—C5	120.2 (2)	N1—C1—H1B	109.5
C10—C7—C5	116.8 (2)	N1—C1—H1C	109.5
C11—C8—C4	118.9 (2)	H1B—C1—H1C	109.5
C11—C8—C5	120.2 (2)	N1—C1—H1D	109.5
C4—C8—C5	120.9 (2)	H1B—C1—H1D	109.5
C13—C9—C4	120.6 (3)	H1C—C1—H1D	109.5
C13—C9—H9A	119.7		

### Hydrogen-bond geometry (Å, °)

**Table:**

*D*—H···*A*	*D*—H	H···*A*	*D*···*A*	*D*—H···*A*
N1—H1A···Cl1	0.90	2.26	3.1345 (19)	163
N1—H1E···Cl1^i^	0.90	2.19	3.0747 (19)	167

Symmetry codes: (i) −*x*+2, −*y*+1, −*z*+2.
